# Successful endoscopic closure using novel clips for a duodenal perforation caused by an endoscopic ultrasound scope

**DOI:** 10.1055/a-2445-8101

**Published:** 2024-11-13

**Authors:** Haruka Nakamura, Noboru Kawata, Junya Sato, Hiroki Sakamoto, Hiroyuki Ono

**Affiliations:** 1 38471 Division of Endoscopy, Shizuoka Cancer Center, Shizuoka, Japan

Iatrogenic duodenal perforation is a serious complication of endoscopic procedures. Surgical intervention is commonly used to manage such perforations, with endoscopic intervention only recommended immediately post-perforation 1 . Conventional endoclips are usually inadequate to completely close large perforations 2 . The over-the-scope (OTS) clip system (Ovesco Endoscopy AG, Tübingen, Germany) has limited availability and is technically complex and expensive.

The MANTIS Clip (Boston Scientific, Marlborough, Massachusetts, USA) is a novel through-the-scope (TTS) clip that is reopenable and rotatable; the sharp claws were designed to grasp tissue securely, overcome the slippage issues of conventional clips, and allow closure of large defects ( Fig. 1 ). They have previously been used for closure of defects after endoscopic resection 3 4 . Herein, we report the use of this novel clip to perform successful endoscopic closure of a duodenal perforation caused by an endoscopic ultrasound (EUS) scope ( Video 1 ).

**Fig. 1:**
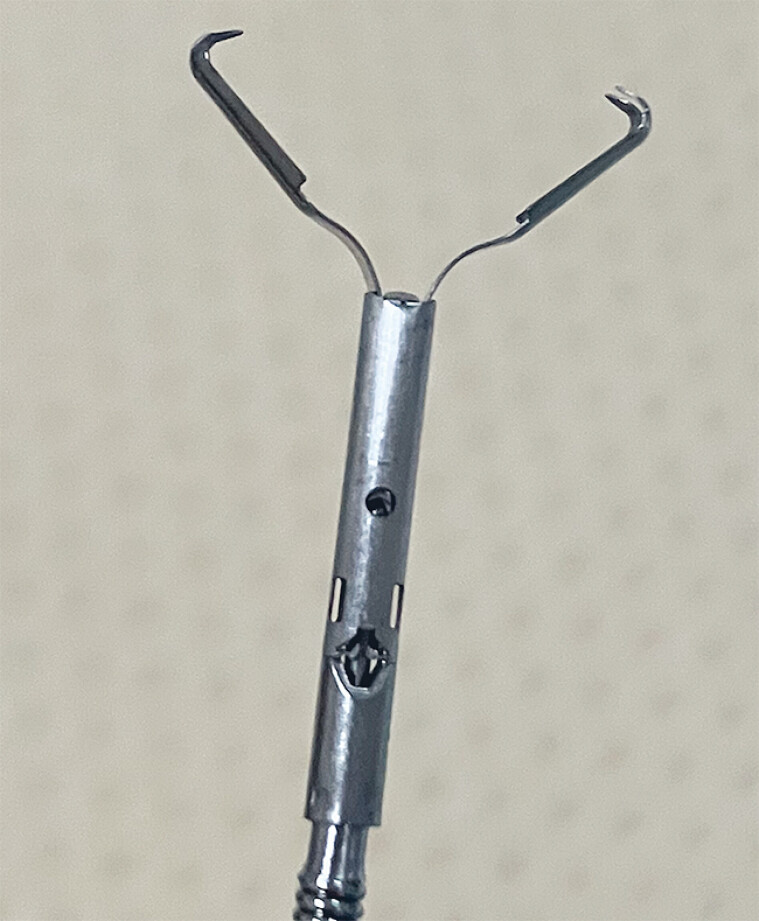
Photograph showing the tip of a MANTIS Clip (Boston Scientific, Marlborough, Massachusetts, USA), which has sharp claws, like the forelegs of a mantis.

Endoscopic closure of a duodenal perforation caused by insertion of an endoscopic ultrasound scope using novel reopenable through-the-scope clips.Video 1

An 82-year-old woman underwent EUS-guided tissue acquisition of a pancreatic head mass. Perforation occurred during the insertion of the EUS scope from the duodenal bulb to the second portion. The EUS scope was immediately removed, and a forward-viewing endoscope was inserted, which confirmed an approximately 10-mm perforation at the superior duodenal angle ( Fig. 2 **a, b** ). Because of the size of the perforation, we used a MANTIS clip to attempt to close it. The normal mucosa at the proximal edge was grasped and pulled towards the anal edge using endoscopic manipulation. The clip was slowly reopened and the two edges were approximated. After confirming that both edges had been securely grasped, the clip was deployed. This procedure was repeated three times, and complete closure of the perforation was achieved in a total closure time of 10 minutes ( Fig. 2 **c** ). Subsequently, the patient’s clinical course was uneventful, with oral fluid intake resuming after 2 days and discharge 7 days post-perforation.

**Fig. 2:**
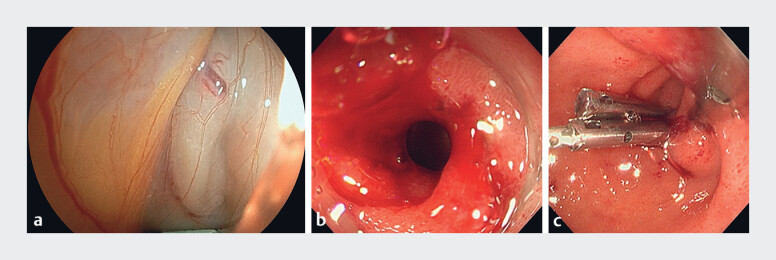
Endoscopic images showing: **a, b** an approximately 10-mm perforation that occurred during insertion of an endoscopic ultrasound scope; **c** the appearance after complete closure using three of the novel reopenable through-the-scope clips.

This novel clip could be a technically simple and effective option for the endoscopic closure of iatrogenic perforations.

Endoscopy_UCTN_Code_TTT_1AO_2AI

## Notes

Endoscopy E-Videos https://eref.thieme.de/e-videos
*E-Videos* is an open access online section of the journal *Endoscopy* , reporting on interesting cases and new techniques in gastroenterological endoscopy. All papers include a high-quality video and are published with a Creative Commons CC-BY license. Endoscopy E-Videos qualify for HINARI discounts and waivers and eligibility is automatically checked during the submission process. We grant 100% waivers to articles whose corresponding authors are based in Group A countries and 50% waivers to those who are based in Group B countries as classified by Research4Life (see: https://www.research4life.org/access/eligibility/ ). This section has its own submission website at https://mc.manuscriptcentral.com/e-videos .
